# Toward Game-Based Digital Mental Health Interventions: Player Habits and Preferences

**DOI:** 10.2196/jmir.6906

**Published:** 2017-04-20

**Authors:** Regan Lee Mandryk, Max Valentin Birk

**Affiliations:** ^1^ Interaction Lab Department of Computer Science University of Saskatchewan Saskatoon, SK Canada

**Keywords:** computer games, mental health, depression, anxiety

## Abstract

**Background:**

Designers of digital interventions for mental health often leverage interactions from games because the intrinsic motivation that results from game-based interventions may increase participation and translate into improved treatment efficacy. However, there are outstanding questions about the suitability (eg, are desktop or mobile interventions more appropriate?) and intervention potential (eg, do people with depression activate enough to play?) of games for mental health.

**Objective:**

In this paper, we aimed to describe the presently unknown relationship between gaming activity and indicators of well-being so that designers make informed choices when designing game-based interventions for mental health.

**Methods:**

We gathered validated scales of well-being (Beck’s Depression Inventory [BDI-II], Patient Health Questionnaire [PHQ-9], trait anxiety [TA], and basic psychological needs satisfaction [BPNS]), play importance (control over game behavior: control; gamer identity: identity), and play behavior (play frequency, platform preferences, and genre preferences) in a Web-based survey (N=491).

**Results:**

The majority of our participants played games a few times a week (45.3%, 222/490) or daily (34.3%, 168/490). In terms of depression, play frequency was associated with PHQ-9 (*P*=.003); PHQ-9 scores were higher for those who played daily than for those who played a few times a week or less. Similarly, for BDI-II (*P*=.01), scores were higher for those who played daily than for those who played once a week or less. Genre preferences were not associated with PHQ-9 (*P*=.32) or BDI-II (*P*=.68); however, platform preference (ie, mobile, desktop, or console) was associated with PHQ-9 (*P*=.04); desktop-only players had higher PHQ-9 scores than those who used all platforms. Platform preference was not associated with BDI-II (*P*=.18). In terms of anxiety, TA was not associated with frequency (*P*=.23), platform preference (*P*=.07), or genre preference (*P*=.99). In terms of needs satisfaction, BPNS was not associated with frequency (*P*=.25) or genre preference (*P*=.53), but it was associated with platform preference (*P*=.01); desktop-only players had lower needs satisfaction than those who used all platforms. As expected, play frequency was associated with identity (*P*<.001) and control (*P*<.001); those who played more had identified more as a gamer and had less control over their gameplay. Genre preference was associated with identity (*P*<.001) and control (*P*<.001); those who played most common genres had higher control over their play and identified most as gamers. Platform preference was not associated with control (*P*=.80), but was with identity (*P*=.001); those who played on all devices identified more as a gamer than those who played on mobiles or consoles only.

**Conclusions:**

Our results suggest that games are a suitable approach for mental health interventions as they are played broadly by people across a range of indicators of mental health. We further unpack the platform preferences and genre preferences of players with varying levels of well-being.

## Introduction

### Motivating Digital Interventions for Mental Health

The prevalence of mental illness is on the rise [1]—the National Institute of Mental Health reports that 18% of adults in the United States had a diagnosed mental illness in 2014 [2]; however, 57% of adults with mental illness in the United States do not receive treatment [3]. Untreated mental illness has serious consequences [4]. The cost of depression and anxiety alone is estimated at US $1 trillion per year [5]. In addition to these financial costs, people experience costs to their well-being that range from a lower quality of life [6] to a loss of life [7]—mental illness is the most important risk factor for suicide as more than 90% of people who commit suicide have a mental or addictive disorder [8,9].

There are several reasons why treatment of mental illness has not evolved to meet the growing demand. First, health care systems cannot handle the burden—there are not enough trained professionals to provide treatment to those in need [10]. Second, access to treatment is lower for people who live in underpopulated areas. For example, in Nunavut (a territory in northern Canada), the suicide rate is 10 times higher than in the rest of Canada [11,12], prompting Nunavut’s chief coroner to suggest that a state of emergency regarding mental illness in the north should be declared [12]. Third, access to treatment is lower for people who live in low-income areas [10]; one in five American adults with mental illness report that the cost of treatment is a barrier for them [3]. And fourth, because mental illness ranges from mild to disabling impairment [1], there are subclinical populations with mild impairment who would benefit from treatment but lack access to a system that is already heavily burdened from treating those with more severe problems.

These limitations in access to treatment cannot be addressed solely by growing the existing mental health system [13]; rather, the solution requires a variety of approaches, including a fundamental shift in delivery mechanisms that will necessarily involve digital solutions [14-16]. There are already several examples of digital systems for interventions in the domain of mental health. For example, previous research has shown that mobile phone apps can be used to treat a variety of mental health disorders such as depression or anxiety [17-19]. Self-help resources such as mobile mental health apps are available for individuals experiencing mild to moderate anxiety and depression [20]. The US Department of Veteran Affairs recommends the mobile app “PTSD coach” (US Department of Veterans Affairs, 2011) that allows people with post-traumatic stress disorder (PTSD) to get access to information about PTSD, track symptoms, and includes small tasks to help people handle their symptoms of stress [21]. The self-help app “Koko” (Koko Inc, 2015) takes a different approach by connecting people in need of emotional support with people who are willing to respond to such a demand. Koko was evaluated in a randomized control trial (RCT) and was shown to be effective [22]. Computerized cognitive behavioral therapy (CBT) has also shown promising results in combating anxiety [23-25], PTSD [21,26,27], and depression [28,29].

Results such as these demonstrate that technology-based mental health interventions offer promise for use in self-help or as an adjunct to clinical treatment [30]; however, there are also several problems that arise when moving digital mental health interventions out of the clinic and into the world. For example, digital solutions can involve interaction with a therapist or formal setting [27,31]; however, many digital solutions are intended to be used without aid from a therapist, which can result in a loss of accountability for a participant [32]. Moving out of the clinic also necessarily results in a loss of external regulation [33] for patients, as there is no longer a psychotherapist but instead a digital app guiding a participant through an exercise. Designers of digital self-help mental health interventions must compensate for these types of problems, as the efficacy of treatment cannot be sacrificed for the wider reach that digital solutions provide.

One solution is to introduce external regulation into digital interventions. For example, we could design systems that require check-ins or provide tangible benefits for daily participation [34,35]. Another solution is to highlight and reinforce the positive benefits to mental health that will eventually occur through sustained participation in the intervention [35,36]. The form of motivation used by these examples, that is, engaging in an activity (training) because it leads to a desirable but separable outcome (benefits to mental health, tangible rewards), is called *extrinsic motivation* [33,37]. Rather than including methods of external regulation or increasing extrinsic motivation, we could alternatively make the treatment activity engaging enough so that people participate because they actually enjoy the treatment itself, and not just because they await the beneficial outcome to their mental health that will result from sustained participation. This form of motivation, that is, engaging in an activity because it is inherently interesting, is referred to as *intrinsic motivation* [33]. Intrinsically motivated people have been shown to be more willing to invest effort into a task and also derive more enjoyment from it [38]. However, the question remains on how treatment activities for improved mental health can be made inherently enjoyable, rather than being undertaken because participants desire the benefits to mental health that sustained participation will eventually provide.

### Game-Based Digital Interventions for Mental Health

One common approach to increasing intrinsic motivation with a digital system is to increase the inherent enjoyment of the activity itself by leveraging the motivational pull of digital games [39]. People play a lot of games; recent estimates suggest that more money is spent purchasing games (US $92 billion) than music (US $18 billion) and movies (US $62 billion) combined [40]. It was found that 4 out of 5 American households own a device that is used to play video games and 115 million Americans play games [41]. Internationally, the global game market is expected to exceed US $102 billion by 2017 [42]. Although people sometimes assume that it is highly immersive console and desktop games that drive the game industry, 35% of those same revenues are expected to be generated through mobile phones and tablets, which people use to play games that are more casual in nature [42]. With so much time and money being spent on digital game play, researchers have started to question what it is about games that make them so compelling to play. One leading explanation for game enjoyment arises out of self-determination theory (SDT) [38] and explains that playing digital games satisfies our psychological needs of competence, autonomy, and relatedness during play itself [39], leading to enjoyment of the experience. Digital game researchers have started to deconstruct how needs satisfaction during play leads to game enjoyment for different players [39,43,44] and in varying gameplay scenarios [45-47]. Others have explored how to leverage these game design elements to motivate people in nongame contexts—a process called gamification [48,49]. Gamified systems have been evaluated in contexts such as learning [47] and behavior change [50,51], and there are also already several examples of gamified interventions in the context of health [52-58]. One of the most successful examples of gamified health interventions is Re-Mission [54], a game designed for children with cancer in which they could shoot cancer cells, manage nausea and constipation, and overcome bacterial infections. A large international study demonstrated that children who played Re-Mission exhibited better adherence to a treatment protocol, improved self-efficacy, and greater cancer-related knowledge than a group who played another computer game [59]. More recently, the application of gamification to treatment has extended beyond addressing physical health into the domain of improving mental health.

For example, a common technique used in CBT is attentional retraining of a participant’s attention away from maladaptive cognitive processes [60]; attention-bias modification training (ABMT) has been shown to be an effective technique to shift a participant’s attention away from negative stimuli [61]. Dennis and O’Toole [52] showed the efficacy of a mobile phone ABMT game in reducing the player’s response to threatening stimuli. In another example, SPARX is a fantasy role-playing game (RPG) for managing depression that is also based on CBT and was shown to be as effective as therapist-administered CBT for treating adolescents with depression [55]. Similarly, CBT principles were translated into biofeedback-supported mini-games in the serious game, Dojo. In a RCT conducted through the school system, adolescents with anxiety who played Dojo showed decreases in anxiety-related symptoms [57], although the study’s commercial control game showed similar effects. Rather than replacing CBT, some game-based approaches instead augment traditional CBT, for example, adolescents with anger-management problems were better able to improve their trait anger when the biofeedback game RAGE-Control was added to a traditional CBT protocol [53]. Finally, the biofeedback game, MindLight, was tested with children with anxiety problems and showed decreases in symptoms over a 3-week trial, although the control game used in the study showed similar effects [58]. A recent meta-analysis of game-based interventions for depression suggests a moderate effect size of game interventions at posttreatment [62]. Furthermore, the study also revealed that games based on entertainment, virtual reality exposure therapy, and exercise showed stronger results than those based on psychoeducation and training.

In an even broader context, games offer an opportunity for improving mental health at a large-scale as it is known that people use games to recover from noxious moods—a process known as mood management. Mood repair through escapism is included in validated scales on both motivations for play [63] and game addiction [64]. In addition, a recent study using gamer forums supports the idea that playing games helps players regulate their mood [65], and children have also noted that a major motivation for play is because games help them relax and forget their problems [66]. On the basis of this idea that games help promote mood equilibrium, several researchers have started to experimentally examine how games help people recover from the stress of life. Using mood management theory, Reinecke [67] used survey data to demonstrate that people use games for recovery from stressful and boring life events. Furthermore, an experiment confirmed that a game that requires interaction facilitates recovery [68]. Similarly, Bowman and Tamborini examined task demand in video games for recovery and showed that greater task demand in a game is better for mood repair, so long as the demand is not too high [69], and that participants choose games with the appropriate level of demand for recovery [70]. Finally, several researchers have suggested that digital games provide an opportunity for improved emotion regulation [71], for example, by rewarding players who successfully downregulate negative affect [72].

### Contribution of This Paper

It is clear that there are potential benefits of motivating participation in digital interventions for mental health by introducing game-based elements; however, there are several outstanding questions about the whether or not games are really appropriate in this context and how great their intervention potential truly is in this domain. For example, we may argue that games have a high intervention potential because people who have low need satisfaction experience greater compulsion to play games [73,74] and are also more likely to experience less control over their play behavior [64]; and because low need satisfaction is related to depression, we may assume that people with depression pass significant time playing games. However, people who have moderate to severe depression find it difficult to activate behaviors [75,76]; simply getting out of bed can be a major challenge. Thus, it is unclear whether people with depression are in fact activating enough to play games. Furthermore, games are argued to have high intervention potential because they are regularly played on mobile phones or other mobile devices, which are platforms that have been targeted for digital interventions in mental health [52,77]. However, are gamers with mental health issues part of the growing group who play games on a mobile platform [41,42] or are they more likely to play in the comfort of their homes on a desktop or console platform? Finally, most game-based interventions use simple game mechanics that are found in casual games, that is, “games with a low barrier to entry that can be enjoyed in short increments” (p.9; [78]), rather than those found in more complex or immersive games, such as RPGs (eg, Mass Effect) or first-person shooter (FPS) games (eg, Call of Duty). If players with depression avoid casual games instead preferring the types of complex and immersive games that are generally played on dedicated gaming consoles, they may eschew playing a game with simple mechanics on a mobile phone and thus may not be motivated to play a game-based mental health intervention delivered on that platform.

Taken together, these questions highlight a problem in our understanding of how games can be used to create digital interventions in mental health: *as researchers, we don’t know how to design, personalize, or market games for mental health interventions, because we don’t know the gaming habits, platform preferences, or genre preferences of people who have poor mental health, low life-satisfaction, or who suffer from depression or anxiety.*

To investigate the intervention potential of digital games, we gathered survey data from 491 online participants who completed validated scales related to their well-being, including on their levels of depression (Beck’s Depression Inventory [BDI-II], Patient Health Questionnaire [PHQ-9], their trait anxiety [TA]), and their satisfaction of their basic psychological needs satisfaction (BPNS). We also asked questions about the importance of play in their lives, including about the control they felt over their gaming behavior and how much they identified as a gamer. Finally, we asked questions about play behavior, including their frequency of play, platform preferences, and genre preferences. Our results suggest that games are a suitable approach for mental health interventions as they are played broadly by people across all indicators of mental health. Throughout the remainder of this paper, we further unpack the play frequency, platform preferences, and genre preferences of players with varying levels of well-being.

## Methods

### Research Questions

Our study was designed to answer several research questions about the intervention potential and suitability of games for mental health interventions. Specifically:

RQ1: Are people with depression or anxiety activating enough to play games? How frequently do they play?

RQ2: Are gamers with depression or anxiety part of the growing group who play games on a mobile platform or are they more likely to play in the comfort of their homes on a desktop or console platform?

RQ3: Do players with depression or anxiety play casual games, or do they prefer more complex and immersive genres of games?

### Participants, Deployment Platform, and Procedure

We recruited 491 participants through Amazon Mechanical Turk (AMT), which acts as a broker between requesters who offer Human Intelligence Tasks (HITs) and paid workers who complete them. Participants received compensation of US $2.5 for their participation, which took approximately 15 min. Ethical approval was obtained from the University of Saskatchewan Behavioral Research Ethics Board, and participants were asked to provide informed consent at the beginning of the task. To comply with ethical guidelines, the task was only available to workers from the United States who were older than 18 years. Additionally, only workers with an approval rate above 90% were offered the task as a means of quality control.

### Measures

We gathered indicators of well-being, indicators of the importance of play, and information regarding gaming behaviors.

#### Indicators of Well-Being

*Degree of depression* was measured using the BDI-II [79] and the PHQ-9 [80]. Both are well-established measures that are commonly used as screening instruments in a clinical context.

The BDI-II is a psychometric test that measures depression using 21 questions that address categories of behavior that are associated with depression, including pessimism, past failure, self-dislike, suicidal thoughts, crying, agitation, and loss of interest in sex. For each question, participants were asked how they have been feeling over the last two weeks, including today. Responses range from not experiencing a feeling or displaying a behavior, for example, “I do not feel sad,” or to an extreme expression of the same feeling or behavior, for example, “I am so sad or unhappy that I can’t stand it.” Responses are then mapped to a severity rating between 0 and 3. Responses are added into a total score (Cronbach alpha=.947); the BDI-II also provides threshold values that indicate categories of depression.

The PHQ-9 asks participants to rate the frequency of negative experiences, over the last two weeks (eg, “Little interest or pleasure in doing things,” “Feeling bad about yourself or that you’re a failure or have let yourself or your family down”) on a scale from “Not at all” to “Nearly every day.” Each rating was assigned a value from 0 to 3. All items were added to create a single score reflecting an overall level of depression (Cronbach alpha=.903).

TA was measured using the 20-item trait scale from the State-Trait Anxiety Scale (STAI; [81]). Participants rated their general distress using statements that people have used before to describe themselves, for example, “I’m calm,” using a 4-point Likert scale from “Not at all” to “Very much” (Cronbach alpha=.951).

*Need satisfaction* was measured using the BPNS scale [82]. BPNS includes subscales for the basic satisfaction of competence (Cronbach alpha=.698), autonomy (Cronbach alpha=.858), and relatedness (Cronbach alpha=.865), as three ongoing needs that people need satisfied to optimally develop and function. Participants rated their agreement to statements, “People I know tell me I am good at what I do” on a 7-point Likert scale from “Strongly Disagree” to “Strongly Agree.”

#### Indicators of Importance of Play

To measure how much importance gaming has in a person’s life, we measured *control over play behavior* (CPB) using the game addiction scale, which assesses excessive, compulsive, and generally problematic use of videogames, and includes subscales for salience (Cronbach alpha=.775), tolerance (Cronbach alpha=.750), mood modification (Cronbach alpha=.840), relapse (Cronbach alpha=.799), withdrawal (Cronbach alpha=.869), conflict (Cronbach alpha=.827), and problems (Cronbach alpha=.770). Participants rate the frequency for which they show a behavior, for example, “Did you play longer then intended,” on a 5-point scale from “never” to “very often.” A higher score indicates greater loss of CPB. Although we were not interested in assessing addictive videogame playing on a clinical level, the scale assesses the degree of uncontrollable excessive and compulsive use of digital games’ that results in social or emotional problems. Only at the extreme end of the scale we would consider the degree of playing as pathological. Our goal was to use the full range of the scale to assess the degree of control over obsessive or harmful game play.

Additionally, we measured *gamer identity*, asking participants to rate how much they self-identify as a gamer on a scale from “Not at all” to “Gamer,” on a single-item scale. Correlations with time played (ρ=.425, using Spearman rank correlation), and CPB (*r*=.417), suggest that the item measures the intended construct. We have previously used this single item alongside a 6-section (60 questions) scale based on self-attributes [83] and found that the 1-item measure correlated at *r*=.735 with the longer version (see Multimedia Appendix 1).

#### Measures of Play Behavior

We gathered subjective measures of play habits and preferences.

*Frequency of play* was measured by asking participants how often on average they play games. Responses were restricted to the following categories: “Every day,” “A few times per week,” “Once per week,” “A few times per month,” “Once a month,” “A few times per year,” “Once per year,” “Not at all.” We grouped players into 3 groups according to play frequency: daily players (n=168), a few times per week (n=222), and once a week or less (n=100).

**Figure 1:**
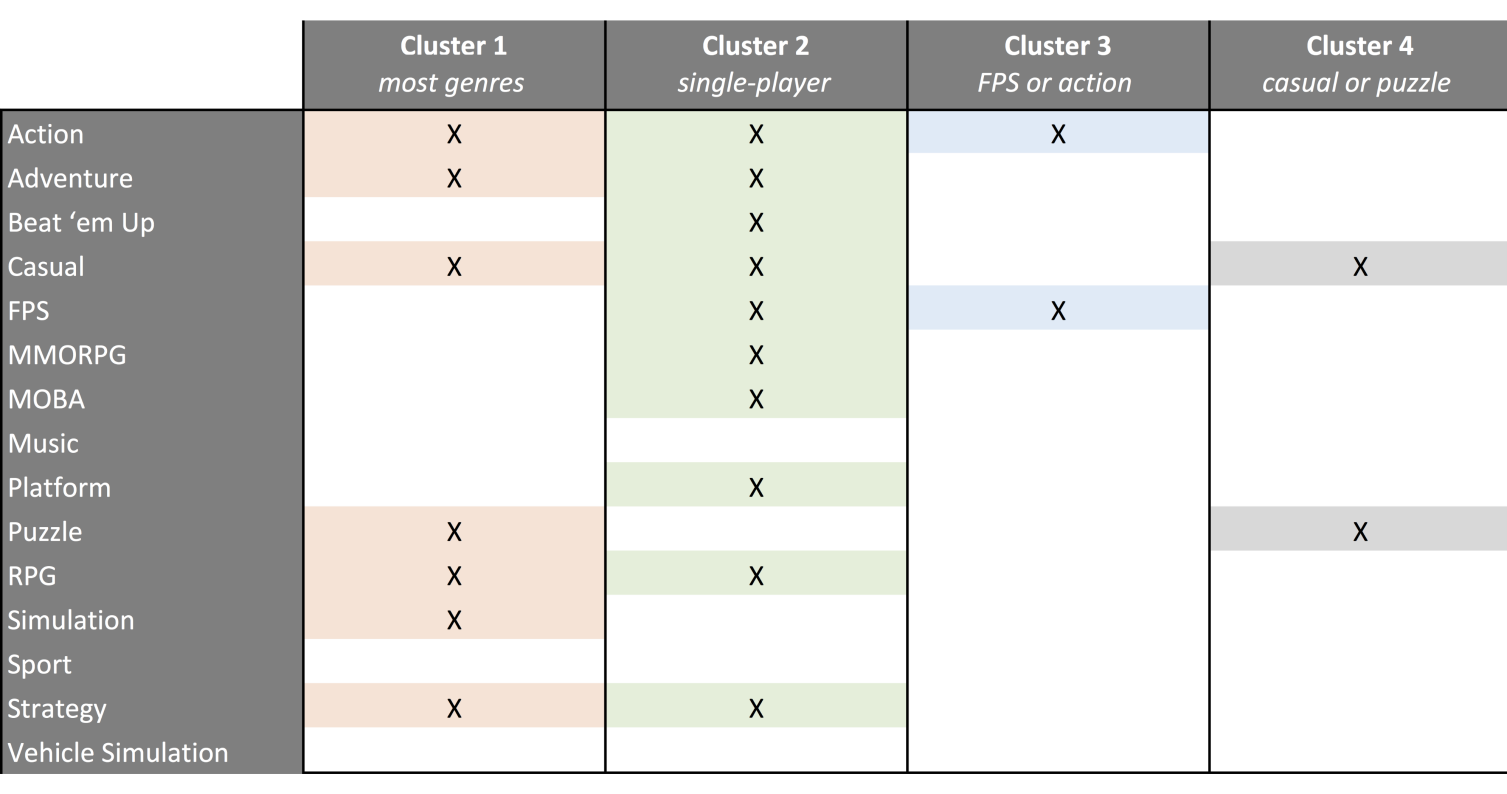
Clusters of genre preferences from the K-means clustering.

Participants indicated their *genre preferences* using a check-all-that-apply question. We asked participants whether or not they enjoy playing the following genres: “action games,” “platform games,” “FPS” “Beat ‘em up,” “adventure games,” “RPG,” “massively multiplayer online role-playing games (MMORPG),” “simulations,” “vehicle simulations,” “strategy games,” “music games,” “sport games,” “multiplayer online battle arena (MOBA),” and “casual games.” Our data shows that some genre categories, for example, music games, had very few true answers, whereas other groups, for example, FPS, had many members. To cluster participants into groups that represent genre preferences, we performed K-means clustering [84] on the 15 genre variables that contained true or false responses. We found the best differentiation at 4 clusters. One cluster included players who enjoy many of the most common genres (n=102); one was for players who mainly enjoy single-player games (n=117); one cluster represented players who enjoyed only FPS and action games (n=150); and one cluster was for players who enjoyed only puzzle and casual games (n=121); see Figure 1.

Participants indicated their *platform preferences* using a check-all-that-apply question. We asked whether participants enjoy playing on “mobile,” “desktop,” or “console” systems. Using K-means clustering on the three Boolean variables, players were separated into 4 categories of platform preference; there was one category for each individual platform (mobile only: n35; console only: n=38; desktop only: n=214) and a combined category for players who use all gaming platforms (n=203).

### Statistical Analysis

#### Data Exclusion

Although AMT has been shown to be reliable as a recruitment tool for research [85] in the domain of human-computer interaction [86] and mental health [15,87], we followed recommendations for excluding participants from the analysis if they showed indications of having not completed the questionnaires with care and attention. We calculated variance for each participant within each survey subscale and removed participants (n=1) from subsequent analyses who demonstrated response variance greater than three standard deviations above mean variance (over the sample) on three or more questionnaire subscales. Having high variance within a subscale is indicative of not paying attention to the survey questions and the reverse-coded items. After the outlier participant was excluded, 490 participants remained (58.2% female (285/490), mean age 34.4 years, SD 11.3) in all of our analyses.

#### Data Analyses

**Figure 2:**
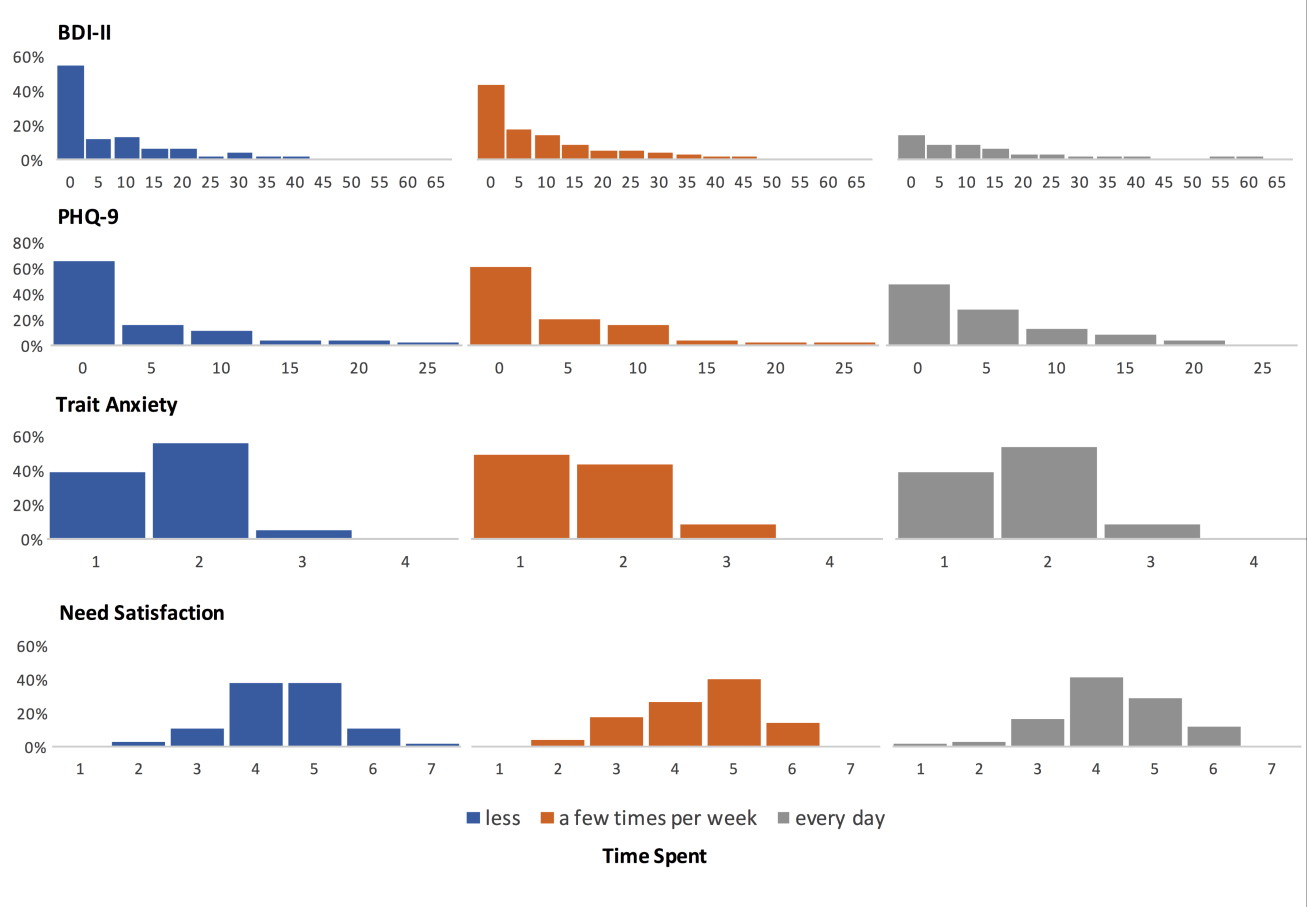
Bar charts of the constructs of well-being (Beck’s Depression Inventory [BDI-II], Patient Health Questionnaire [PHQ-9], Trait Anxiety, and Need Satisfaction) displayed for each level of time spent (less, a few times per week, every day). For each construct, the chart shows the count per bin on the x-axis, represented as a percentage of participants in that level of time spent.

BDI-II, PHQ-9, and TA data were summed into a single score reflecting level of depression, and TA respectively. As shown in Figure 2, data for BDI-II and PHQ-9 followed a Pareto distribution, thus we log-transformed data for both scales to increase robustness of the subsequent analyses [84]. For BPNS, we calculated the mean across the three subscales of satisfaction of competence, autonomy, and relatedness, into a single score representing need satisfaction (Cronbach alpha=.925). For CPB, we calculated the average across all subscales; however, we removed the mood modification subscale, because it showed low factor loadings in the original construction of the scale [64] and because playing games as a method of dissipating noxious moods is a common behavior that allows people to recover from stress or boredom [67,88] and represents a beneficial coping strategy rather than a pathological behavior [67] (Cronbach alpha=.947).

To investigate the relationship between mental health and play behavior, we performed multivariate analysis of variance (MANOVA), with play behavior variables (play frequency, platform preference cluster, genre preference cluster) as factors, and indicators of well-being (BDI-II, PHQ-9, STAI trait, BPNS) and importance of play (control, identity) as dependent measures, controlling for sex and age. It is important to note that the predictor variables are not randomly assigned, but reflect the choices of participants. Therefore, our results cannot be interpreted as a causal relationship between the factor and the measure (as is common with ANOVA interpretation), but rather as an indicator of a statistically significant relationship between a categorical variable and a continuous variable. All analyses were performed using SPSS 24 (IBM Corp).

## Results

### Sample Distribution

**Figure 3:**
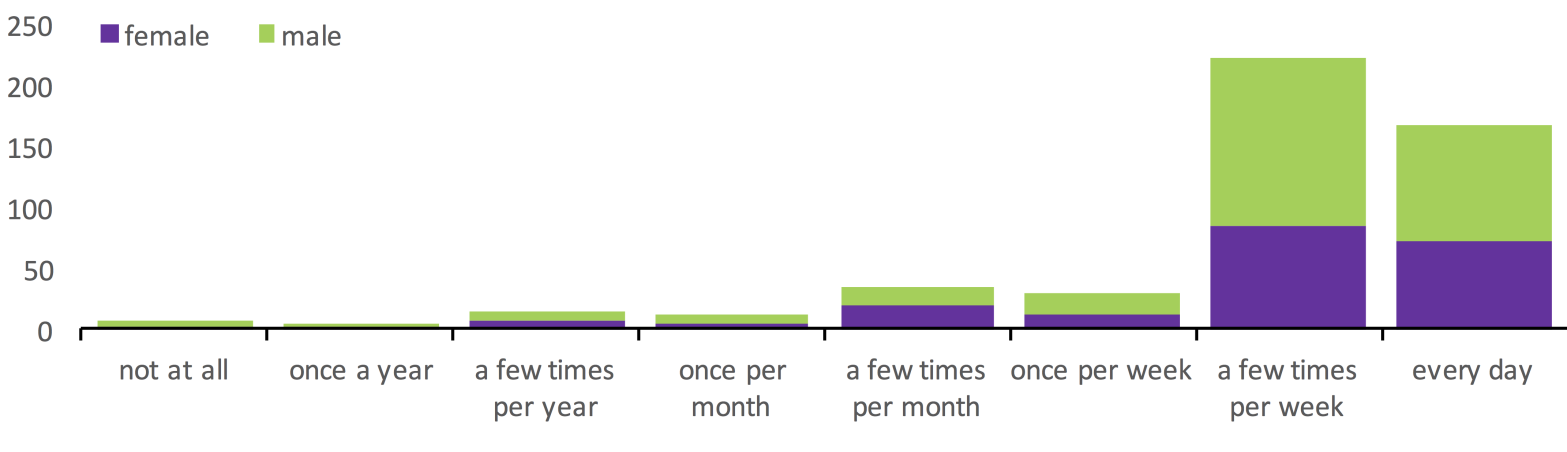
Stacked bar chart of frequency of play and gender.

Figure 3 shows the distribution of responses to the question on the frequency of play. As shown, the vast majority of respondents played games at least a few times per week or every day. Furthermore, as shown in Figure 3, there was no systematic relationship between gender and game play frequency (a Kruskal-Wallis test of game play frequency on gender was nonsignificant: χ^2^_2_=3.8, *P*=.15).

**Figure 4:**
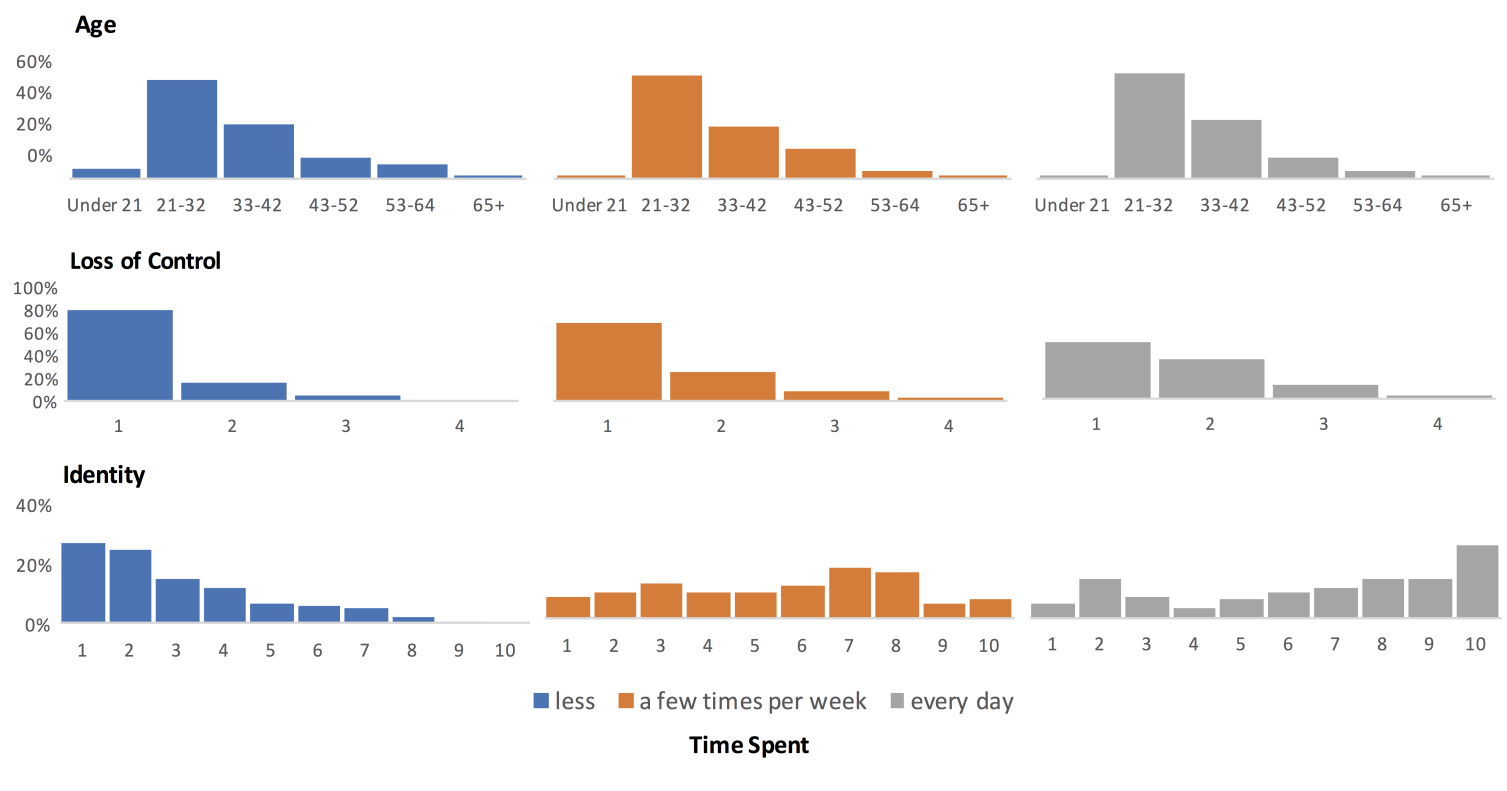
Bar charts of the constructs of age, loss of control, and identity displayed for each level of time spent (less, a few times per week, every day). For each construct, the chart shows the count per bin on the x-axis, represented as a percentage of participants in that level of time spent.

The age of our sample ranged between 18 and 73 years (mean 34.1, SD 9.8); furthermore, as Figure 4 shows, there was no systematic relationship between age and game play frequency (a Kruskal-Wallis test of game play frequency on age was nonsignificant: χ^2^_2_=1.2, *P*=.55).

**Figure 5:**
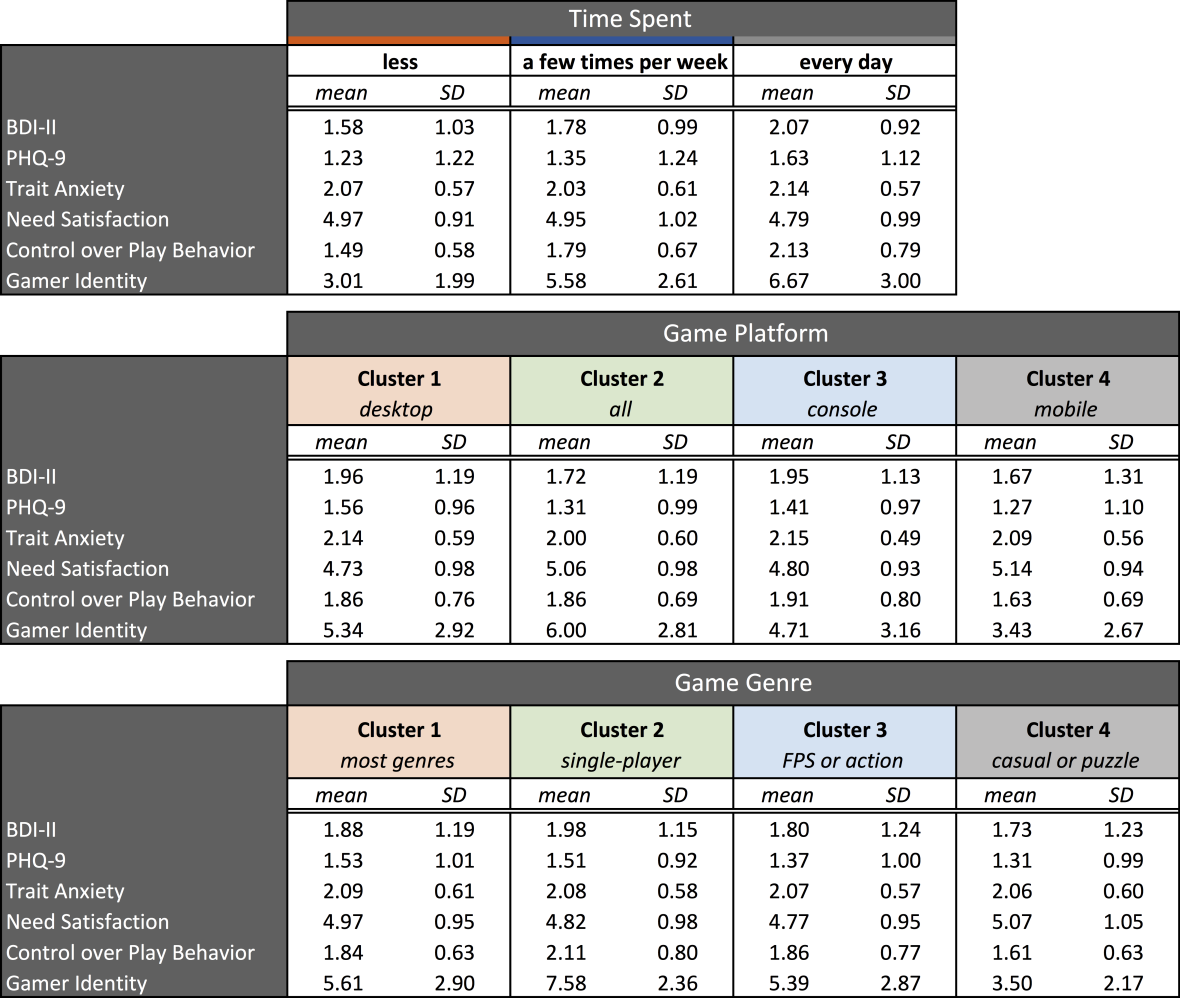
Means and standard deviations (SD) for the indicators of well-being and importance of play for: (Top) frequency of play groups, (Middle) platform clusters, and (Bottom) genre clusters.

Figure 2 shows the distributions of responses for the indicators of well-being for the three frequencies of play groupings. Figure 5 shows the means and standard deviations for the indicators of well-being and importance of play separately for the frequency of play groups, platform clusters, and genre clusters.

### Correlations Among Dependent Measures

There were significant correlations between the dependent measures. As expected, CPB was correlated with gamer identity (*r*=.42). Furthermore, BDI-II was positively correlated with PHQ-9 (*r*=.88; they both measure mental health) and TA (*r*=.70), and negatively correlated with need satisfaction (*r*=−.66). PHQ-9 was positively correlated with TA (*r*=.71), and negatively correlated with need satisfaction (*r*=−.67). TA was negatively correlated with need satisfaction (*r*=−.79).

### Importance of Play

Frequency of play showed a significant relationship with CPB (*F*_2,485_=26.48, *P*<.001, *η*^2^_p_=.098), and gamer identity (*F*_2,485_=61.00, *P*<.001, *η*^2^_p_=.20). Bonferroni-corrected post hoc tests showed that frequency of play differed between all levels of CPB (all *P*<.01). The more people play, the more loss of control they experience; and on all levels of gamer identity (all *P*<.01), the more people play, the more they identify as a gamer.

In terms of genre preference, we found significant relationships with CPB (*F*_2,484_=6.35, *P*<.001, *η*^2^_p_=.038), and gamer identity (*F*_2,484_=30.56, *P*<.001, *η*^2^_p_=.159). For CPB, Bonferroni-corrected post hoc tests revealed that playing across common genres was related to a loss of CPB compared with playing (1) action or FPS-games or (2) casual or puzzle-games. There was no difference between single player games and the other three categories (all *P*>.05). Bonferroni-corrected post hoc tests for gamer identity showed the highest identity ratings for those who played all common genres, compared with the other three clusters (all *P*<.001). The single player and action or FPS clusters also showed higher identity ratings compared with the puzzle or casual-cluster (both *P*<.001), which was the genre cluster with the lowest overall ratings of gamer identity.

Platform preference showed a significant effect on gamer identity (*F*_2,484_=5.69, *P*<.001, *η*^2^_p_=.03); but not on CPB (*P*=.80). Bonferroni-corrected post hoc analysis revealed that people who play on all devices identify more as a gamer, compared with people who play on mobile (*P*<.001), or console (*P*<.001) only. There was no difference between all devices and desktop (*P*=.09).

### Indicators of Well-Being

#### Measures of Depression

Frequency of play showed a significant relationship with PHQ-9 (*F*_2,485_=5.78, *P*=.003, *η*^2^_p_=.023), and BDI-II (*F*_2,485_=5.35, *P*=.01, *η*^2^_p_=.022). For PHQ-9, Bonferroni-corrected post hoc tests revealed that people who play every day score significantly higher on the PHQ-9 compared with those who play less than a few times per week (*P*<.001), or play a few times per week (*P*<.001). For BDI-II, Bonferroni-corrected post hoc tests revealed that people who play every day score significantly higher than people who play less than a few times per week (*P*<.001). There was no significant relationship between genre preference and PHQ-9 (*P*=.32) or BDI-II (*P*=.68).

Furthermore, when we consider just those players who are classified as having significant depression (n=35) according to the BDI-II [79], we find that 12 play every day, 16 play a few times per week, and 7 play once per week or less.

Platform preferences showed a significant relationship with PHQ-9 (*F*_2,485_=2.79, *P*=.04, *η*^2^_p_=.017), but not BDI-II (*P*=.18). Bonferroni-corrected post hoc tests revealed that people who used only a desktop computer scored higher on the PHQ-9 (*P*=.03) than people who use all devices.

#### Measures of Anxiety

TA shows no significant relationship with frequency of play (*P*=.23), platform preference (*P*=.07), or genre preference (*P*=.99).

#### Measures of Need Satisfaction

Need satisfaction showed no significant relationship with frequency of play (*P*=.25) or genre preference (*P*=.53). For platform preference, however, we see a significant relationship (*F*_2,485_=4.16, *P*=.01, *η*^2^_p_=.025) with need satisfaction. Bonferroni-corrected post hoc tests revealed that people who play only on desktop computers score significantly lower on need satisfaction than those who play on all devices (*P*=.004). The mobile and console clusters showed no differences compared with the other clusters.

## Discussion

### Principal Findings and Comparison With Prior Work

In summary, our results for importance of play showed that people who play frequently identify more as a gamer and feel less in control over their play behavior. It is not a surprise that investing more in an activity makes this activity a bigger part of our life and is integrated more into our identity [83]. Additionally, we show that people who play across many genres—as opposed to a small set of closely related genres—identify more as gamers and experience less control over their play behavior. Furthermore, we show that desktop players identify more as gamers than players who use only mobile or console devices.

In terms of the effects on indicators of well-being, we showed that playing every day was associated with higher scores on the BDI-II and PHQ-9, but did not affect TA or need satisfaction. It is not surprising that playing every day is associated with indicators of poorer mental health, as research on pathological gaming has found that low need satisfaction is associated with the experience of game addiction [64]. In addition, research on disordered patterns of play has found that people with low basic satisfaction of relatedness feel more compelled to play (ie, plan to play more in the future) but get less enjoyment out of the play experience [73].

Furthermore, desktop-only players scored higher on the PHQ-9 and lower on need satisfaction; however, genre preference was not significantly related to any indicator of well-being. This last result bodes well for intervention designers who wish to incorporate game-based elements into their digital interventions, as it appears that they are not limited in terms of the appeal of different game mechanics or genres and can use what works best for their particular intervention design. However, the finding that playing on desktop computers alone (as opposed to consoles, mobile devices, or all three) is associated with indicators of poorer mental health suggests that intervention designers might want to account for this preference when deciding on a platform for delivery.

### Implications for the Design of Digital Interventions for Mental Health

In terms of the research questions that we set out to answer:

RQ1. Are people with depression or anxiety activating enough to play games? How frequently do they play?

The majority of our participants played games a few times a week or daily. In addition, PHQ-9 scores were higher for those who played daily than for those who played a few times a week or less, and BDI-II scores were higher for those who played daily than for those who played once a week or less.

*Our sample suggests that people with depression are activating enough to play games at least a few times per week, and often daily.*

RQ2. Are gamers with depression or anxiety part of the growing group who play games on a mobile platform or are they more likely to play in the comfort of their homes on a desktop or console platform?

Depression and low need satisfaction were both significantly associated with desktop-only play.

*Although players with mental health issues use all types of platforms for playing games, there seems to be an increased prevalence of desktop-only play for people with depression that designers may want to consider when creating game-based interventions for mental health.*

RQ3. Do players with depression or anxiety play casual games, or do they prefer more complex and immersive genres of games?

Genre preferences were not associated with any indicators of mental health.

*Our results suggest that designers can choose from a range of game mechanics and genres in creating game-based interventions for mental health, and should not feel limited to a specific genre or type of game.*

### The Benefits of Games for Mental Health Interventions

Our results suggest that games may be a valuable approach for the design of digital mental health interventions. Here, we discuss three main advantages of game-based intervention design for mental health based on our results and previous work [89].

#### Motivational Pull

We motivated the contribution of this paper by arguing that leveraging the motivational pull of games [39] may increase participation in a digital mental health intervention. For efficacious treatment, patients need to adhere to the intervention repeatedly over the long-term, and previous work has shown that participants who engage in training under their own volition exhibit better adherence, which may translate into greater efficacy [90]. In our previous work, we have sought out ways to use interaction design to increase intrinsic motivation with digital apps. We initially showed that how we see our own personality in a game (game-self) affects the motivation to play [91]. We then leveraged this finding to show that identification with an in-game avatar in a simple game leads to increased engagement with and invested effort in a boring task, also translating into significant differences in motivated behavior as measured by time spent in a free-choice task [92]. Additionally, we demonstrated that these motivational benefits lasted in a daily training task over the medium term (1 week) [34]. Together, this work demonstrates how we can use games to foster intrinsic motivation with apps and foreshadows how game-design could be used to facilitate longer-term engagement in interventions for the treatment of mental health issues by fostering intrinsic motivation.

#### Broad Appeal

Although people from different demographics may be more susceptible to mental health issues (eg, adolescents [93], lower socioeconomic status [3]), the prevalence of depression, anxiety, or personality disorders are not restricted to a specific demographic [94]; people of various ages, genders, socioeconomic statuses, and cultures are affected. Similarly, although games may have a reputation of appealing to the young male demographic, the appeal of digital games extends across a range of demographics. Our own sample in this study (n=490) shows how age and gender are not related to frequency of play. The Entertainment Software Association (ESA) [41] reports that although 26% of gamers are aged less than 18 years, 27% are over 50 years. Additionally, 44% of gamers are female—in fact, females over 18 years represent a significantly larger part of the game-playing population (33%) than boys less than 18 years (15%) [41].

Our previous research on computer games spans various ages of players, ranging from a focus on children with developmental disabilities [95] to the elderly who live in institutionalized care [96]. Although we sometimes focus our research on committed players who self-identify as gamers [97] or play specific games [98,99], our use of crowdsourcing platforms (such as AMT used in this study) to gather data and conduct experiments allows us to access a broad and representative sample of people who enjoy playing a diverse range of digital games [34,43,92]. In creating game-based interventions for mental health issues, designers do not need to be concerned that their solutions will only appeal to a narrow group of people with specific gaming interests. Our results from this study suggest that the motivational pull of games described in the previous section applies broadly across the range of demographic groups who can benefit from apps that aim to improve mental health.

#### Accessibility

Because mental health issues are prevalent across demographic groups, it is important to ensure that people have access to treatment, independent of their work schedule, geographic location, or the capacity of the health care system. Games are generally accessible, independent of time of the day or location (thanks to the increased prevalence of mobile games). Because digital content can be delivered to any place that has Internet access, games are accessible in most geographical locations, which gives them advantages over traditional psychotherapy for countries with distributed and remote populations, such as in Nunavut—the territory in northern Canada that has a suicide rate 10 times greater than the rest of the country [11,12]. Although games are accessible in remote locations, they also hold advantages for addressing mental health in populated regions in which the capacity for treatments is exhausted. The shortage of available treatment resources can result in greater waiting time for patients [100] and undiagnosed mental issues in early stages [101], which may translate into tangible (eg, loss of work hours) and intangible (eg, loss of life quality) outcomes. Game-based interventions may help to bridge this gap. Finally, the convenience of in-home therapy offered by game-based approaches (as opposed to visiting a clinic) may expand the reach of mental health interventions, making treatment more accessible for those who need it [95].

### Limitations

Our results demonstrate several important relationships between the habits and preferences of players and indicators of their mental health. However, there are several limitations that should be addressed through future work. First, the survey uses self-report measures of play behavior and is subject to all the biases present in self-report. Although the indicators of mental health also use self-report, the measures that we used are standard in terms of diagnosing depression (ie, BDI-II) and are recommended by the World Health Organization for assessing mental health (ie, the PHQ-9). Second, the survey was only available to be taken by the US residents, and thus our results are not generalizable beyond this particular context. Third, our results provide information on the play habits and preferences of people with varying levels of depression, anxiety, and need satisfaction to provide guidance to developers of digital interventions for mental health. Because we do not randomly assign people to experimental conditions, there is no intention in this paper of claiming causal links between game play habits and mental health. Research on pathological gaming [102-104] addresses the relationship between play and mental health from that perspective; our goal was to establish that people with depression and anxiety activate enough to play games from a variety of genres, and on a range of platforms. Finally, we only examined how much people played and what their play preferences were in terms of platform and genre. It is possible that there are other factors regarding the context of play that are also important to understand, for example, Lemola et al [103] showed how habitual computer game playing at night (between 10 pm and 6 am) is associated with an increase in depression scores, even after controlling for the total time played, suggesting that the timing of play is also relevant in this context. In future work, we will investigate the patterns of play in addition to the habits and preferences of players.

### Conclusions

In this paper, we use data from a Web-based survey (N=491) to describe the relationships between gameplay habits, gameplay preferences, and indicators of mental health by answering research questions about the differential gameplay habits of people with varying levels of depression, anxiety, and need satisfaction. In general, we reveal that the vast majority of people in our sample play games a few times a week or more, and that playing daily is associated with indicators of depression. Desktop play was also associated with higher indicators of depression and lower need satisfaction. As expected, those who played more had identified more as a gamer and had less control over their gameplay.

Our results suggest that games are a suitable approach for mental health interventions as they are played broadly by people across a range of indicators of mental health, have strong motivational pull, and are accessible to players from a broad range of demographics. Our contribution is of interest to the community as we establish that games are an approach with great potential to add to the growing literature on digital interventions for improving mental health.

## Appendix

Multimedia Appendix 1 Description of one-item measure of gamer identity.

## Abbreviations

ABMT: attention-bias modification training
AMT: Amazon Mechanical Turk
ANOVA: analysis of variance
BDI-II: Beck’s Depression Inventory
BPNS: basic psychological needs satisfaction
CBT: cognitive behavioral therapy
CPB: control over play behavior
ESA: Entertainment Software Association
FPS: first-person shooter
HIT: Human Intelligence Task
MANOVA: multivariate analysis of variance
MMORPG: massively multiplayer online role-playing game
MOBA: Multiplayer online battle arena
PHQ-9: Patient Health Questionnaire
PTSD: post-traumatic stress disorder
RCT: randomized controlled trial
RPG: role-playing game
SDT: self-determination theory
STAI: State-Trait Anxiety Scale
TA: trait anxiety
US: United States
