# Supplementary material for: Toward Game-Based Digital Mental Health Interventions: Player Habits and Preferences
Source: J Med Internet Res. 2017 Apr 20;19(4):e128. doi: 10.2196/jmir.6906 (PMC5418528; doi:10.2196/jmir.6906)
Supplement: Multimedia Appendix 1 [file jmir_v19i4e128_app1.pdf]

Multimedia Appendix 1

To validate our one-item measure of gamer identity, we compared results from that single question to a previously-evaluated instrument for measuring identity. We gathered a sample of 180 people, collecting the Self-Attribution Questionnaire (SAQ, [83]) and our one-item measure of gamer identity, next to other demographic variables. The SAQ measures attitudes people have about aspects of their identity, e.g., intelligence, physical ability, or musical ability. Each attribute was assessed on six different scales, i.e., the rating of the attribute relative to others, the certainty about the rating of relative attribute, the importance of the attribute, the discrepancy between the attribute and one's ideal self - the person someone wants to be, how someone else would assess the attribute, and the desire to have the attribute assessed by someone else. To assess gamer identity, we added "video game ability" to the self-attribution scale. A Pearson correlation between the average of all SAQ scales (60 items) and self-identification as a gamer shows a medium to strong correlation ($r=.735$), indicating that gamer identity explains 54% of the variance in self-attributed game ability.
